# Payments to Hospitals by Insured In-Patients without Dependants

**Published:** 1915-09-25

**Authors:** 


### NATIONAL INSURANCE ACT DEVELOPMENTS.

### Payments to Hospitals by Insured In-Patients Without Dependants.

When the National Health Act first came into operation it was hoped that the voluntary hospitals might benefit by payments made by insured persons treated by them. Such patients are in possession of funds to provide for their maintenance when disabled by sickness, and the proposition that whilst in hospital they should pay over these funds to the institution seemed, on the face of it, worthy of consideration. But when one looked closely into the matter it was found necessary to divide the insured, for the purposes of such consideration, into two classes—*i.e.*, those with dependants and those without.

The maintenance of the breadwinner while away from the home, plus the cost of the subsistence of his family, would together undoubtedly be more than the expenses were all residing under one roof. This fact, added to the supposition that sickness benefit is intended for the sustenance of the dependants of the insured person, as well as himself, would make the act of taking away the benefit for the use of a hospital appear hard-hearted to such an extent that a contribution could not be asked for in the absence of evidence of other substantial form of support.

The insured person without dependants is in an entirely different position, for if the hospital provides his maintenance without charge, it is equal to giving him a present of money, because he would be allowed to retain cash received for the specific expenditure that the hospital undertakes.

There are, of course, exceptions to every rule, and a regulation affecting a large number of people must press hard upon a few. Whatever steps a hospital may decide to take in respect of these payments, discretion as to enforcement must be given to the officer responsible for the work of the department dealing with assessment and collection. Now and again an insured patient without dependants must be considered exempt or be accepted at a reduced rate. There is, for instance, the patient who has no legal dependants, but whose circumstances are such that there are people who have as strong a moral claim upon him as if they had a right to the definition. Again, there is the patient who is discharged as incurable, and has to face the world with a total disablement benefit of 5s. a week. To take the whole of the accrued sickness benefit in such a case, where there are no other means, would be cruel, and quite out of accordance with the sympathetic nature of the voluntary system.

### The Practical Rule.

In order to ascertain to what extent the practice of drawing upon these resources prevails in hospitals, inquiry has been made at a number of representative institutions, and the result of this investigation may be summed up by saying that the matter is most often left to the good feeling of the patients. Inquiry is certainly made in most cases as to ability and willingness to contribute, and where it is evident that no hardship is involved, an invitation is given by the almoner, or other responsible officer, for a contribution to be made. As the Irishman said, this invitation is sometimes a " cordial invitation," but the matter is left at that.

Here and there the thing is taken a little further. At one or two institutions the in-patient (without dependants) is asked to sign a letter to the Approved Society asking that the sickness benefit shall be paid to the hospital. The Approved Society may honour such a letter or may not; it certainly is not obliged to do so unless it has specifically declared its willingness to enter into an agreement in accordance with the provisions of the National Health Act. This point we shall touch upon later. Otherwise the letter may hit the mark, and it may not; all depends upon the good will of the Society to which it is addressed and the knowledge and subsequent acts of the patient.

The degree of good will between Approved Societies and a hospital varies considerably. Some accept generously the fact that it is all to the advantage of a Society to obtain the assistance of the skilled treatment of a member by a hospital. It is obvious that hospital treatment will often shorten the period under which sickness benefit is allowed, and will occasionally save the payment of total disablement benefit. So clear is this contention that it is difficult to believe that Approved Societies require further education on the subject.

### What Influences the Approved Society.

A large and powerful Society, when this proposition was put to it a few weeks ago, expressed the opinion that a Society would not raise any objection to payments to hospitals but for the risk of offending and losing its members; that the competition for new members is so keen that the Society paying most in benefits to its members is likely to be more successful than its competitors.

But the National Health Act makes clear provision for payments to hospitals. Under Subsection (*c*) of Section 12 payment is allowed to be made direct to a hospital if the inmate is a member of an Approved Society, has no dependants, and there is an agreement for the purpose between the Society and the hospital (not the inmate and the hospital).

There are a number of Societies ready to enter into such an agreement. Amongst these are the following, some of which it will be seen are Societies known to have a large number of members:—

National Deposit Friendly Society.
Domestic Servants' Insurance Society.
National Union for Insurance.
Provident Association of Warehousemen, Travellers, and Clerks.
Nurses' Insurance Society.
National Amalgamated Approved Society.
Wiltshire Working Men's Conservative Benefit Society.

There are probably many more, and if our readers will supply additional names we will publish an extended list later, as the information is of considerable interest to hospital administrators.

### Text of the Agreement.

The usual form of agreement is the following:—

AGREEMENT made the..............day of................. one thousand nine hundred and fifteen between............... ..................of.......................for and on behalf of the ......................................Approved Society, hereinafter called "The Society," of the one part, and.................... ..........................................for and on behalf of the ........................................, hereinafter called "The Hospital," of the other part, whereby it is agreed as follows:—

In consideration of the hospital maintaining and treating or continuing to maintain and treat...................... of.................................................., a member of the Society, being a person with no dependants within the meaning of the National Insurance Act, 1911, as an inmate of the Hospital, the Society agree to pay the Hospital as from the..............day of.................the sum of..........................per week during such period as the said......................................shall continue to be an inmate of the said Hospital, and the Hospital agree to apply the sums so paid to them under this agreement towards the maintenance of the member in the said Hospital.

AS WITNESS our hands this..........................day of ..........................one thousand nine hundred and fifteen.

On behalf of the Society.........................................

On behalf of the Hospital.........................................

### An Additional Clause.

Some Societies will add to this a clause that, if it is found on the discharge of a member that it is necessary to furnish the member with any surgical instrument or further surgical treatment, the Society may first charge the cost of such surgical instrument or special treatment to the moneys held in respect of the benefits, and after such charge is met the residue only shall be payable to the hospital. This it is probable few will object to.

### Counsel's View.

But the above form of agreement is not the only possible one within the meaning of the Act. To return to the system of obtaining the signature of a patient to a letter to a Society, it should be said that Section 3 of the Act quite clearly prohibits assignments of charges and benefits, and this prohibition is qualified only by the sections quoted above. However, we have before us a legal opinion that if an Approved Society enters into a definite agreement with a hospital that it will make the payments to the hospital in the case of an inmate who has signed a letter of authority, such an agreement would be effectual and would bind an inmate once he had signed it so that he would not be able to revoke it. This opinion goes on to say that unless there is such an agreement the mere writing of a letter, even if in fact acted upon by a Society, would not be valid or effectual, and the letter could at any time be withdrawn by the inmate, and it might be found that a hospital might legally be compelled to repay sums it had already received in respect of the inmate.

There is one Society (one of the few largest in the country) which is willing to make an agreement covering these letters of authorisation and request issued by members, and arranges (a considerable source of saving of time and trouble) to make the resulting payments from the head office instead of through its numerous agents throughout the country. The form of letter in use is as follows:—

To The Secretary,

..............................Approved Society.

Dear Sir,

*Membership No.*...............

I beg to inform you that I have this day become an in-patient at the..............................Hospital, and I authorise and request you to pay to the Secretary of the Hospital the amount due to me for any sickness benefit while I am resident in the Hospital.

Yours truly,

(Signature of Member).....................................

Home address .....................................

.....................................

This letter may be signed in advance of the date of admission by the patient, but must be dated on the day of entrance to the wards.

### A Reservation.

A curious qualification to this variation of the agreement allowed by the Act, is that should a patient die in the hospital no payment will be made to the institution by the Society. It is claimed that this reservation is authorised by a ruling of the National Health Commissioners.

Acting on the lines covered by these agreements one hospital in London has, without a single case of hardship to an insured patient, succeeded in obtaining payment in thirty-eight cases since the commencement of the year, being 8 per cent. of the total number of admissions. This institution is favourably placed in respect of time and opportunity to make agreements, as in the majority of cases there is a sufficient period between application and admission to carry through the necessary business. It is, of course, far easier to deal with the matter of payment in this interval than later, but it should not be impossible to enter into an agreement after a patient has been admitted to the wards.

